# Spatial–Temporal Variations, Ecological Risk Assessment, and Source Identification of Heavy Metals in the Sediments of a Shallow Eutrophic Lake, China

**DOI:** 10.3390/toxics10010016

**Published:** 2022-01-04

**Authors:** Xiaomei Su, Hong Ling, Dan Wu, Qingju Xue, Liqiang Xie

**Affiliations:** 1 Jiangsu Provincial Key Laboratory of Environmental Engineering, Jiangsu Provincial Academy of Environmental Sciences, Nanjing 210036, China; suxm@jshb.gov.cn (X.S.); wudan@jshb.gov.cn (D.W.); 2 State Key Laboratory of Lake Science and Environment, Nanjing Institute of Geography and Limnology, Chinese Academy of Sciences, Nanjing 210008, China; qjxue@niglas.ac.cn (Q.X.); lqxie@niglas.ac.cn (L.X.)

**Keywords:** lake sediment, heavy metal, spatial distribution, risk assessment, source identification

## Abstract

The contamination of heavy metals (Pb, Cr, Hg, Cd, Ni, Cu, Zn, As, and Sb) in the sediments were investigated in Lake Yangcheng, a eutrophic lake in China. Results showed that the average concentrations of each metal in the surface sediments generally exceeded their corresponding background values. Higher values were observed in deeper zones, supporting the retention and accumulation of heavy metals in the core sediments. The spatial distributions of metal averages, pollution load index (PLI), and combined ecological risk index (RI) revealed that ecological risks were highest in the west lake, followed by middle lake, and were lowest in the east section. For the temporal variations of metal contents, the highest concentration was usually observed in the winter. However, the seasonal dynamics of Hg showed a different pattern with higher values in the autumn and lower values in the winter. According to contamination factor (CF), the Hg and Sb contaminations were considerable, while the other metals were moderate contamination. In terms of geoaccumulation index (I_geo_) values, sediments were moderately–heavily polluted by Sb and moderately polluted by Hg, Cd, and Ni. Meanwhile, Hg exhibited a considerable health risk, while Cd and Sb were moderate risks, based on single ecological risk index (Er) values. Significant positive correlations among heavy metals and principal component analysis (PCA) indicated that anthropogenic activities were major sources. The source of Sb might be different from other metals, with industrial discharge as the main loading. This study highlighted the urgency of taking measures to prevent Hg, Sb, and Cd pollutions in Lake Yangcheng, especially the west region of this lake.

## 1. Introduction

The contamination of heavy metals has received continuous attention due to their strong toxicity, abundant content, and high frequency in aquatic and terrestrial environments, posing potential ecological risks to the existent of living organisms in lakes [1,2,3,4], rivers [5,6,7], reservoirs [8], oceans [9], and soils [10,11,12], etc. [13]. Highest concentrations were measured in invertebrate species for all metals but Zn. As, Cd, and Cu seemed to concentered more in crabs and shrimp [14]. Heavy metal pollution also poses serious environmental threats to the health of human beings through consumption of contaminated fishery products, which is considered as the consequence of bioaccumulation and biomagnification via food chains or food webs [15,16,17]. Heavy metals are usually derived from natural sources and anthropogenic activities, such as atmospheric deposition, erosion of bedrocks, industrial pollutants, agricultural activities, and sewage discharge [7,18,19]. Regular human activities, such as road traffic, also contribute to heavy metal pollution [20]. Therefore, it is crucial to monitor and analyze the levels and distributions of heavy metals to gain a relatively accurate evaluation of metal contamination in the ecosystems. Besides, it might also be helpful to take more efficient actions to control and reduce the potential ecological risks once the sources of metal pollution are identified.

Lakes are particularly susceptible to the degradation of their ecological value as they provide important sources of drinking water, habitats for various living organisms, and recreational opportunities for humans [21,22]. There are intensive studies demonstrating the spatial and temporal variations of heavy metals and their sources, transportations, adsorption, and desorption, as well as health risk assessment in aquatic ecosystems [1,2,23,24,25]. However, these studies mostly focused on the contamination of heavy metal in the surface sediment. Less information is available about the vertical distribution of heavy metals in different depths as well as their health risks in eutrophic shallow lakes, located in ecologically sensitive regions [3,26,27]. Additionally, few reports are concerned with Sb contamination in the freshwater lakes even though Sb could have a similar toxicity coefficient as As [28,29].

Lake sediment can adsorb and immobilize a certain amount of heavy metals in the water–sediment interface, while it can also be a potential source of contamination when environmental conditions change towards favoring release mechanisms. Therefore, the analysis of heavy metal contamination in the surface sediment and their vertical distribution in the deep layers are useful solutions to study the metal pollution in an individual lake. A variety of indices are proposed to evaluate metal contamination, including contamination factor (CF), geoaccumulation index (I_geo_), pollution load index (PLI), and potential ecological risk index (Er and RI). The wide applications of these indices are helpful to better understand the pollution degree in sediments. Additionally, there are increasingly worldwide reports about the spatial distribution, risk assessment, and source analysis of heavy metals in the lakes [1,2,4,18,23,25,30]. These previous studies provide an opportunity for researchers to better compare heavy metal contamination among similar lacustrine ecosystems.

Lake Yangcheng is a medium-sized shallow lake with a surface area of approximately 120 km^2^, which serves as an important drinking water source for Suzhou city, Jiangsu Province, China. The lake is famous for producing high-quality cultured Chinese mitten crab (*Eriocheir sinensis*, CMC), a well-known food in China [31]. In 2015, a total of 2100 tons of hairy crabs was produced in this area, contributing to an income of approximately 90 million USD. Previous investigations indicated that elevated concentrations of heavy metals were observed in the surface water and sediments of Lake Yangcheng because of metallic wastewater discharges from finishing plants. Meanwhile, a decline of metal concentrations in the lake was reported due to reducing production capacity of the copper mine plant and the construction of two dams in recent years [32]. Unfortunately, there is still a lack of information on the spatial distribution, risk assessment, and source identification of heavy metals in sediments of this eutrophic shallow lake.

In this study, we conducted a comprehensive survey to explore the spatial distributions and temporal patterns of heavy metals in the sediments of Lake Yangcheng. Meanwhile, the vertical distributions of heavy metals with an increase in depth were also examined. In addition, heavy metal contamination was jointly assessed by CF, I_geo_, Er, PLI, and RI, then all the metals were ranked according to their toxic levels and health risks. Finally, the potential sources of heavy metals were also explained by multivariate statistical analyses. Our goal was to provide some scientific support for the management and treatment of heavy metal pollution in this shallow lake.

## 2. Materials and Methods

### 2.1. Study Area and Sample Collection

**Figure 1:**
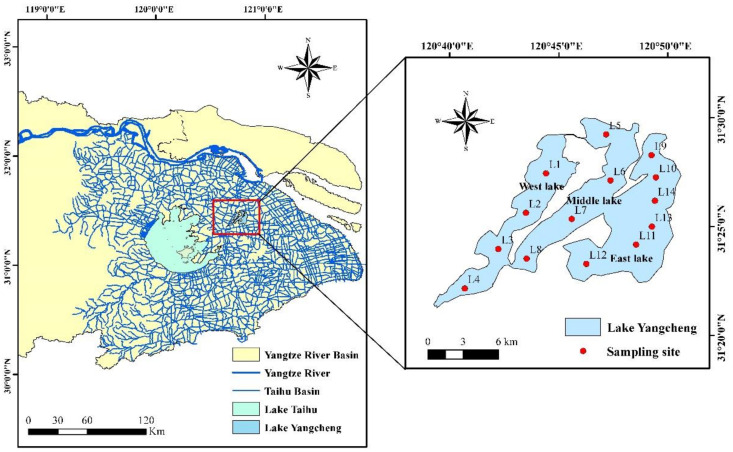
Map of Lake Yangcheng and the locations of sampling sites in this study.

Lake Yangcheng (31°21′–31°30′ N, 120°39′–120°51′ E) is located in the lower Yangtze River Basin and to the northeast of Lake Taihu in eastern China (Figure 1). The lake is a typical shallow lake with an average depth of 2.05 m, stretching for about 17 km from north to south and 11 km from east to west. The catchment has a humid subtropical monsoonal climate, with an annual mean temperature of 15.7 °C and an monthly precipitation of 92.6 mm [33]. Rainfall is mainly concentrated in the period from May to September, contributing to above 60% of the total every year. Another main water supply is from the Yangtze River and Lake Taihu via river systems, most of which originate on the west part of this lake.

Lake Yangcheng is divided into three parts by two nearly parallel narrow strips of land (Figure 1), including west lake (L1, L2, L3, and L4), middle lake (L5, L6, L7, and L8), and east lake (L9, L10, L11, L12, L13, and L14). East lake is the biggest part, with a surface area of 52.5 km^2^, followed by middle lake (34.6 km^2^), and west lake (32.0 km^2^).

Seasonal surface sediment samples (about 0–5 cm) were collected from each sampling site using a Petersen grab sampler in February, May, August, and October of 2018, corresponding to the four seasons, namely winter, spring, summer, and autumn, respectively. After sampling, the sediment samples were sealed in clean polyethylene plastic bags, placed in a cooler, transported to the laboratory within 6 h, and stored at −20 °C for further analysis. Thus, a total of 56 surface sediment samples were obtained in this study. Additionally, in order to explore the vertical distribution of heavy metal in the sediment, sediment cores (about 20 cm) were also collected from 14 sampling sites in February 2018 using a piston corer. Each sediment core was divided into 6 sections from top to bottom and every 3 cm represented a different layer, including 0–3 cm, 3–6 cm, 6–9 cm, 9–12 cm, 12–15 cm, and 15–18 cm, respectively.

### 2.2. Chemical Analysis

All the sediment samples were freeze-dried; then stones and plant fragments were removed and ground by passing through a 200-mesh sieve. About 0.2 g of each sample was completely digested by a HCl-HNO_3_-HF-HClO_4_ system in Teflon beakers. The concentrations of Pb, Cr, Cd, Ni, Cu, Zn, As, and Sb were determined using inductively coupled plasma mass spectrometry (ICP-MS, Agilent 7700×, USA) under standard operating conditions. Hg content was measured by an automated direct Hg analyzer Hydra-C (Leeman Labs Inc., Hudson, NH, USA).

Quality assurance and quality control (QA/QC) measures were confirmed by the analysis of duplicate samples, standard reference materials, and blank control. The concentration of each metal was expressed in mg/kg of dry weight basis. The recoveries for different metals of the standard reference samples ranged from 85 to 115%. The analytical precision for 3 replicates was within ±5%, and the measurement errors between the determined and certified values were less than 5%. The detection limits of Pb, Cr, Hg, Cd, Ni, Cu, Zn, As, and Sb were 0.02, 0.1, 0.002, 0.01, 0.05, 0.02, 0.2, 0.1, and 0.05 mg/kg, respectively.

### 2.3. Assessment of Metal Pollution in the Sediment

#### 2.3.1. Contamination Factor (CF)

The CF was calculated using the following relationship:

(1) $$\begin{array}{c} \mathrm{CF}=\frac{C_{i}}{C_{b}} \end{array}$$

where *C_i_* and *C_b_* are the measured concentrations of an element within a sample and its corresponding background value, respectively. The background values of each heavy metal detected in this study referred to the baseline in the soil of Jiangsu Province, China [34]. The contamination levels were classified into 4 degrees, based on their intensities, on a scale ranging from 1 to 6, namely low degree (CF < 1), moderate degree (1 ≤ CF < 3), considerable degree (3 ≤ CF < 6), and very high degree (CF ≥ 6) [35].

#### 2.3.2. Geoaccumulation Index (I_geo_)

The I_geo_ was globally used to assess metal contamination in the sediment fractions considering the influences of anthropogenic activities, calculated as follows:

(2) Igeo=log2Cik×Bi 

where *C_i_* is the concentration of metal *i* examined in the sediment and *B_i_* is the geochemical background concentration of the given metal. Factor *k* (usually set as 1.5) is the background matrix correction factor due to lithospheric effects. A 7-level classification of I_geo_ was defined as follows: I_geo_ ≤ 0 indicates practically uncontaminated; 0 < I_geo_ ≤ 1 indicates uncontaminated–moderately contaminated; 1 < I_geo_ ≤ 2 indicates moderately contaminated; 2 < I_geo_ ≤ 3 indicates moderately–heavily contaminated; 3 < I_geo_ ≤ 4 indicates heavily contaminated; 4 < I_geo_ ≤ 5 indicates heavily–extremely contaminated; I_geo_ > 5 indicates extremely contaminated.

#### 2.3.3. Pollution Load Index (PLI)

PLI of all the measured metals were calculated according to the following equation:

(3) $$\begin{array}{c} \mathrm{PLI}=\sqrt[{n}]{CF_{1}\times CF_{2}\times CF_{3}\times \ldots \times CF_{n}} \end{array}$$

where *CF_i_* is the contamination factor of a given metal. PLI values were classified into 4 levels: unpolluted with PLI < 1; moderately polluted with 1 ≤ PLI < 2; heavily polluted with 2 ≤ PLI < 3; extremely polluted with PLI ≥ 3 [36].

#### 2.3.4. Single Ecological Risk Index (Er)

Er is used to assess the ecological risk of a single metal and calculated according to the reference (Hakanson, 1980).

(4) Cfi=C0iCni

(5) $$\begin{array}{c} E_{r}^{i}=T_{r}^{i}\times C_{f}^{i} \end{array}$$

where *C_f_^i^* is the pollution coefficient of metal *i*; *C_0_^i^* is the measured concentration of metal *i* in the sediments, and *C_n_^i^* is the corresponding background concentration. E_r_ is the single ecological risk index for a given metal; *T_r_^i^* is the toxic response factor of metal *i* (Pb = 5, Cr = 2, Hg = 40, Cd = 30, As = 10, Ni = 5, Cu = 5, Zn = 1, Sb = 10) (Zhang et al., 2018). According to E_r_, the risk of single metals in sediments were categorized into five levels: (1) E_r_ ≤ 40, low risk; (2) 40 < E_r_ ≤ 80, moderate risk; (3) 80 < E_r_ ≤ 160, considerable risk; (4) 160 < E_r_ ≤ 320, high risk; (5) E_r_ > 320, very high risk.

#### 2.3.5. Combined Ecological Risk Index (RI)

RI is used to assess the combined ecological effects of various heavy metals. To obtain the combined risks of Pb, Cr, Hg, Cd, As, Ni, Cu, Zn, and Sb, RI was calculated as follows:

(6) $$\begin{array}{c} \mathrm{RI}=\sum_{i=1}^{n}E_{r}^{i}\; \end{array}$$

Using the calculated RI values, risks of metals in the sediments were categorized into four levels: (1) RI ≤ 150, low risk; (2) 150 < RI ≤ 300, moderate risk; (3) 300 < RI ≤ 600, high risk; (4) RI > 600, very high risk [35].

### 2.4. Statistical Analysis

The data of the heavy metals in the sediments were expressed as average values and standard deviation (SD), based on pooled samples. The spatial distributions of metal concentrations and their ecological risks were displayed by interpolation maps using ArcGIS (Environmental Systems Research Institute Inc., Redlands, CA, USA). Kruskal–Wallis non-parametric test was used to determine the significance of difference in heavy metals between three lake areas and among four seasons. In order to explore the potential sources of heavy metals, the Spearman correlation analysis was performed among samples given that the variation of several heavy metals did not satisfy the normal distribution. Furthermore, principal component analysis (PCA) was conducted to assess the relationship between different heavy metals in the lake sediment. The data were standardized by a z-scale transformation before PCA to avoid the influence caused by numerical ranges. All statistical analyses were conducted using statistical software package SPSS 20.0. Statistical significance was set as *p* < 0.05.

## 3. Results

### 3.1. Spatial and Temporal Variations of Heavy Metals in the Surface Sediment

**Figure 2:**
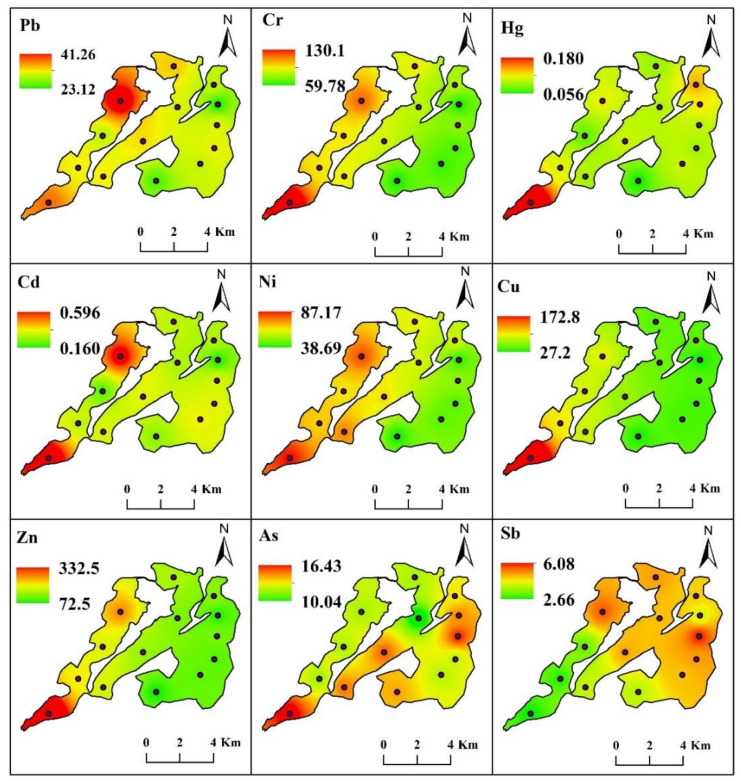
The spatial distributions of nine heavy metals were displayed according to the averages of four seasons in the lake sediment. The colored areas indicate the contents of heavy metals (unit: mg/kg) at the sampling sites. The color spectrum ranges from green (lowest) to red (highest).

The spatial distributions in the concentrations of nine heavy metals showed obvious heterogeneity according to the average values of four seasons (Figure 2). Significant difference (*p* < 0.05) among three lake areas was observed in the majority of metals, except Hg, Cd, and As. In general, the concentrations of most heavy metals detected in this study were highest in the west lake, while As and Sb concentrations were highest in the east lake. The highest concentration of Pb was observed at site L1, which was located in the northern part of west lake. However, for Cr, Hg, Cd, Ni, Cu, Zn, and As, the maxima were all recorded in site L4, which is located in the southwest part of west lake (Figure 2). The spatial distribution of Sb showed a different pattern from other metals, with highest value in the east section and lowest in the west part (Figure 2).

We also explored the seasonal patterns of heavy metals contents in the surface lake sediment (Table 1). There were significant temporal variations in the concentrations of Cr, Hg, Cd, Zn, and As among seasons (*p* < 0.05). While no significant variations were observed in the concentrations of Pb, Ni, Cu, and Sb between different seasons (*p* > 0.05). For all the metals except Hg, highest concentration was usually observed in the winter. The seasonal dynamics of Hg showed a different pattern with higher values in the autumn and lower values in the winter (Table 1).

The average concentrations of heavy metals in Lake Yangcheng (averages of three lake areas) were ranked as a decreasing order in mg/kg, as follows: Zn (135.73) > Cr (82.21) > Ni (59.77) > Cu (54.67) > Pb (30.12) > As (13.64) > Sb (4.38) > Cd (0.316) > Hg (0.090) (Table 2). Generally, the average concentrations of each heavy metal all exceeded their corresponding background baseline values (Table 2). According to the concentrations of heavy metals in different lake areas, higher values were observed in the west lake except for As and Sb. We also compared the concentrations of heavy metal in the surface sediment in the present study with previously published reports in other freshwater lakes. In comparison between lakes, the values of heavy metals in the surface sediment from Lake Yangcheng remained moderate levels. However, the concentrations of Ni in Lake Yangcheng were relatively higher than other lakes in China (Table 2).

### 3.2. Vertical Distributions of Heavy Metals in the Deep Sediment

**Figure 3:**
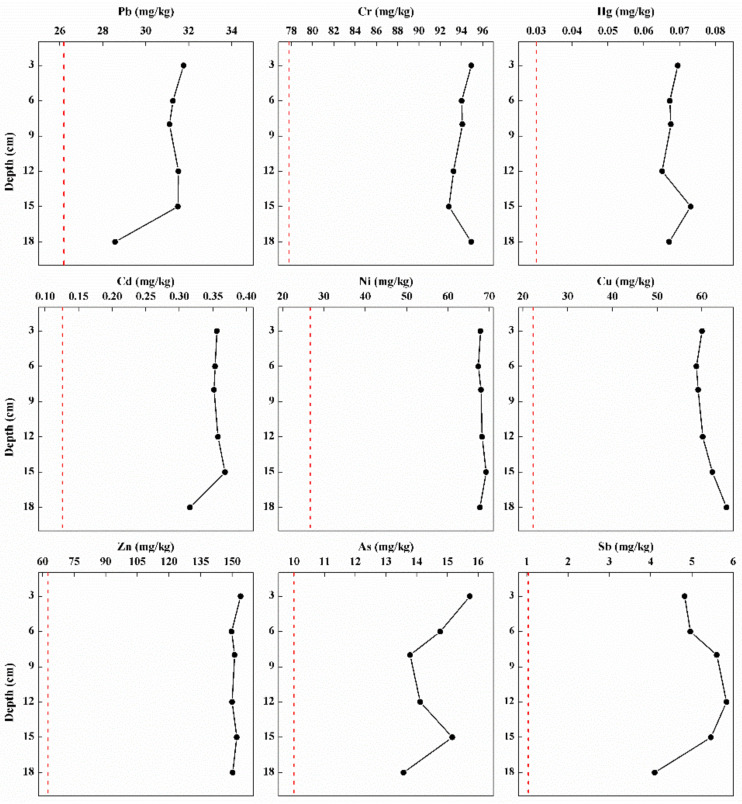
The vertical distributions of heavy metals in the sediment with an increase in depth. Red lines indicated the background baselines for each heavy metal.

The concentrations of heavy metals detected in the deep sediment all exceeded their corresponding background baselines (Figure 3). Furthermore, the average concentrations of heavy metal in the core sediment were above the mean value of that in the surface sediment except Hg (Table 2 and Figure 3). Different patterns of fluctuation were observed in the concentration of heavy metals with an increase in sediment depth (Figure 3). According to the dynamics of metals in the core sediment, Pb, Cd, and Sb concentrations showed a decreasing trend with the increase in depth, whereas there was a sudden increase in layers 15 cm, 15 cm, and 12 cm, respectively. A slight increase was observed for Cr concentrations in layer 15 cm and a minor increase was observed in the variations of Cu concentrations. There was not too much difference in the dynamics of Ni and Zn concentrations in the deep sediment. Additionally, the vertical variations of Hg and As showed strong fluctuations in the deep zone (Figure 3). An increase in RI was observed in the bottom sediment (Figure S1).

### 3.3. Ecological Risks Assessment of Heavy Metals in the Lake Sediments

**Figure 4:**
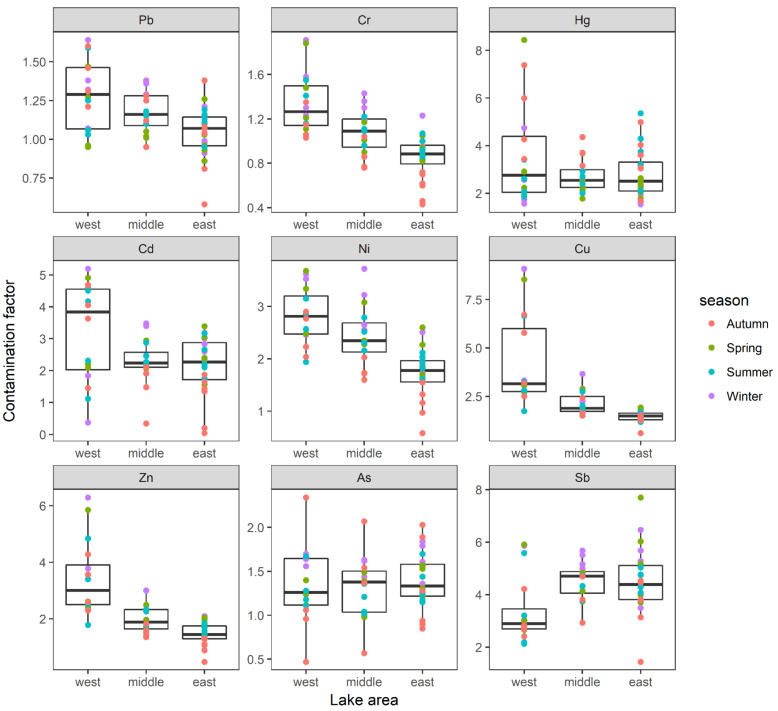
The spatial distributions of CF of each heavy metal were displayed in three lake areas. Boxes represent the 25th to 75th percentiles; straight lines within the boxes mark the median; and the small dots indicate the average values of the 4 seasons.

The values of contamination factor in Lake Yangcheng ranged from 0.58 to 1.61 for Pb, 0.43 to 1.91 for Cr, 1.54 to 8.43 for Hg, 0.04 to 5.19 for Cd, 0.58 to 3.72 for Ni, 0.60 to 9.09 for Cu, 0.47 to 6.29 for Zn, 0.47 to 2.34 for As, and 1.44 to 7.70 for Sb, respectively. The averages of CF were ranked in a decreasing order, as follows: Sb (4.21) > Hg (3.01) > Cd (2.51) > Cu (2.45) > Ni (2.24) > Zn (2.17) > As (1.36) > Pb (1.15) > Cr (1.06). Thus, according to the classification of CF, only Hg and Sb were found to be considerable contamination, while the other seven heavy metals showed a moderate degree of pollution. Furthermore, the spatial distributions of CF showed different patterns for each metal (Figure 4). For the majority of heavy metals, namely Pb, Cr, Cd, Ni, Cu, and Zn, the mean values of CF were highest in the west lake, followed by middle lake, and lowest in the east lake. The contamination of As and Sb were observed to be most serious in east lake (Figure 4).

**Figure 5:**
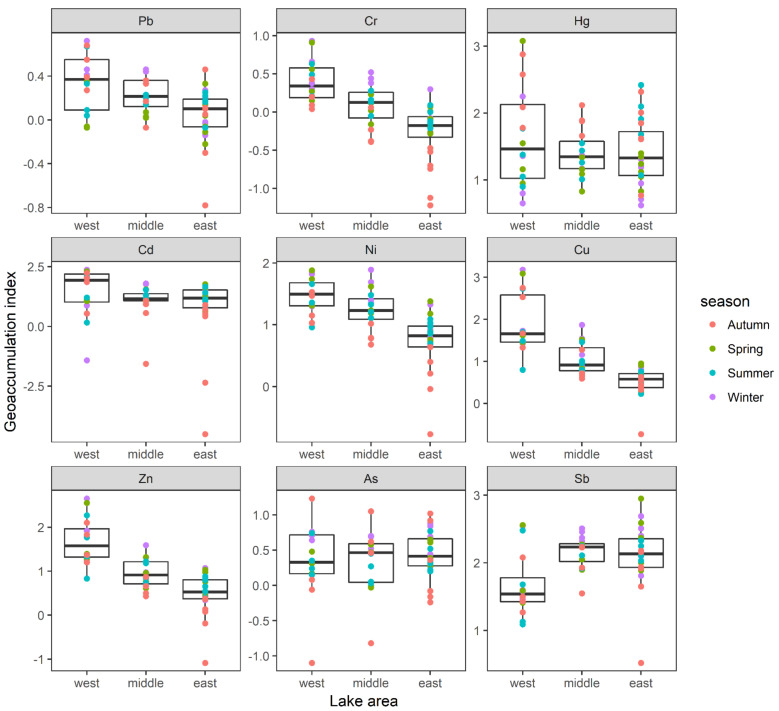
The spatial distributions of I_geo_ of each heavy metal were displayed in three lake areas. Boxes represent the 25th to 75th percentiles; straight lines within the boxes mark the median; and the small dots indicate the average values of four seasons.

I_geo_ values in Lake Yangcheng ranged from −0.78 to 0.72 for Pb, −1.22 to 0.93 for Cr, 0.62 to 3.08 for Hg, −4.51 to 2.37 for Cd, −0.77 to 1.89 for Ni, −0.73 to 3.18 for Cu, −1.09 to 2.65 for Zn, −1.10 to 1.23 for As, and 0.52 to 2.95 for Sb, respectively. The averages of I_geo_ were ranked as follows: Sb (2.01) > Hg (1.47) > Ni (1.09) > Cu (1.07) > Cd (1.06) > Zn (0.96) > As (0.39) > Pb (0.18) > Cr (0.02). Thus, Sb was found to be moderately–heavily polluted. Additionally, Hg, Cd, Ni, and Cu were found to be moderately polluted. While the other four heavy metals, including Zn, As, Pb, and Cr, were found to be unpolluted–moderately polluted according to the annual average of I_geo_ values. Additionally, the spatial distributions of I_geo_ showed different patterns for each metal (Figure 5). For the majority of heavy metals, namely Pb, Cr, Cd, Ni, Cu, and Zn, the mean values of I_geo_ were highest in the west lake, followed by middle lake, and lowest in the east lake. However, the contaminations of As and Sb were more serious in east lake (Figure 5).

**Figure 6:**
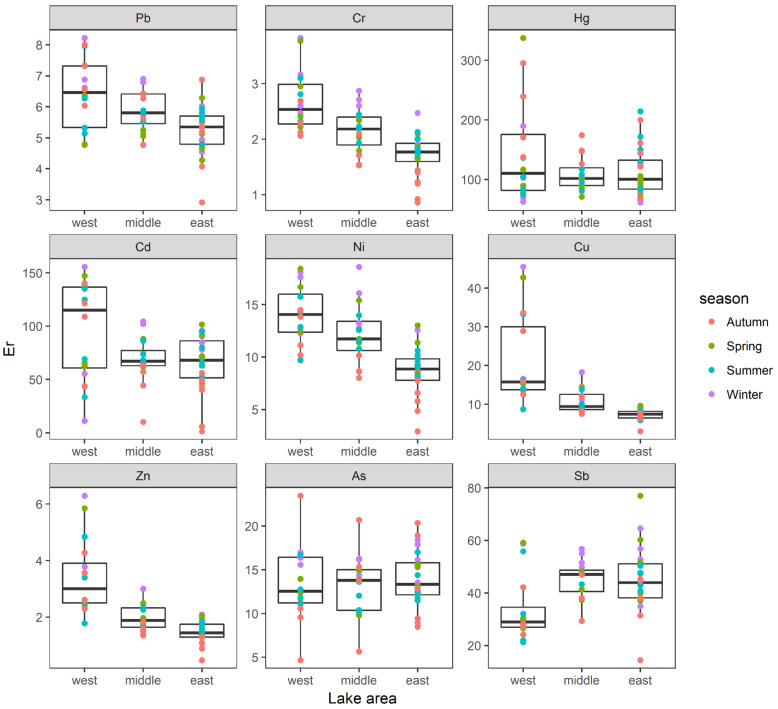
The spatial distributions of Er of each heavy metal were displayed in three lake areas. Boxes represent the 25th to 75th percentiles; straight lines within the boxes mark the median; and the small dots indicate the average values of four seasons.

Er values in Lake Yangcheng ranged from 2.91 to 8.22 for Pb, 0.86 to 3.82 for Cr, 61.47 to 337.23 for Hg, 1.32 to 155.60 for Cd, 2.92 to 18.59 for Ni, 3.01 to 45.43 for Cu, 0.47 to 6.29 for Zn, 4.66 to 23.45 for As, and 14.39 to 77.01 for Sb, respectively (Figure 6). The averages of Er were ranked as: Hg (120.42) > Cd (75.33) > Sb (42.14) > As (13.64) > Cu (12.26) > Ni (11.19) > Pb (5.75)> Zn (2.17) > Cr (2.11). Therefore, Hg was found to be moderate to high risks. Additionally, Cd and Sb were found to be moderate risks. While the other six heavy metals, including As, Cu, Ni, Pb, Zn, and Cr, showed low risks according to the annual average of Er values (Figure 6).

**Figure 7:**
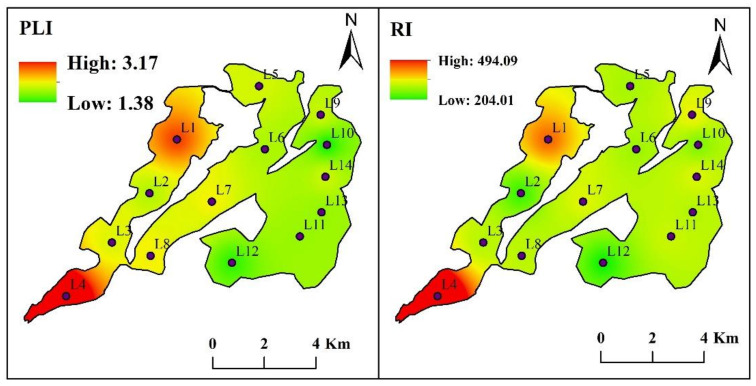
The spatial distributions of PLI and RI in the surface sediment according to the values of 14 sampling sites in Lake Yangcheng. The colored areas indicate risk values at the sampling sites. The color spectrum ranges from green (lowest) to red (highest).

PLI ranged from 1.38 to 3.17 with an average of 1.96, which indicated a moderate pollution in the sediment of whole lake. RI ranged from 107.98 to 602.18 and its average was 285.01, which also indicated a moderate contamination in the entire lake (Figure 7). The spatial distributions of PLI and RI showed similar patterns, with high risks observed in the west lake, followed by middle lake, and low risks occurred in the east lake. The maximum of PLI and RI were both observed at site L4 with values of 3.17 and 494.11, respectively, while the minimum of PLI occurred at site L10 with a value of 1.38 and the lowest RI occurred at site 12 with a value of 204.01 (Figure 7). In terms of seasonal variations of PLI and RI, PLI was ranked as winter > spring > summer > autumn, while RI was ranked as summer > autumn > spring > winter (Table 1, Figures S2 and S3). No significant variations in PLI and RI were observed between seasons (*p* > 0.05) (Table 1).

### 3.4. Source Identification of Heavy Metals in the Lake Sediments

The nine heavy metals showed different correlations with each other (Table 3). Strong positive correlations were observed between Pb and Cr, Pb and Hg, Pb and Cd, Pb and Ni, Pb and Cu, and Pb and Zn (r = 0.373–0.650, *p* < 0.01). Cr showed significantly positive correlations with Cd, Ni, Cu, and Zn (r = 0.642–0.947; *p* < 0.01). Besides, strong positive correlations were also observed between Cd and Ni, Cd and Cu, Cd and Zn, Ni and Cu, Ni and Zn, and Cu and Zn (r = 0.624–0.938, *p* < 0.01). Hg merely showed a slight positive correlation with Pb (r = 0.373; *p* < 0.01). Sb only showed positively significant correlation with Cd (r = 0.310; *p* < 0.05). However, As showed no correlation with any other heavy metals in this study (Table 3).

Two components were extracted from the PCA with a combined explanation of 72.96% of the total variance (Table 4). The first principal component (PC1) accounted for 55.30% of the total variance with high loadings of Zn (r = 0.965), Cr (r = 0.932), Cu (r = 0.897), Ni (r = 0.89), Cd (r = 0.87), and Pb (r = 0.751). We speculated that Zn, Cr, Cu, Ni, Cd, and Pb originated from similar sources, in view of their strong correlations with each other. The second principal component (PC2) explained 17.66% of the total variance with high loading of Sb (r = 0.924). It seemed that anthropogenic activities, such as industrial wastewater, were the major sources of Sb.

## 4. Discussion

### 4.1. Spatial–Temporal Variations of Heavy Metals in the Sediment

Lake Yangcheng serves as an important drinking water source, a tourist attraction, and a local aquaculture region for producing high-quality hairy crabs in southeast China [31]. Therefore, it is vital to have a comprehensive understanding of the ecological risks posed by heavy metals in this eutrophic shallow lake. Surprisingly, the concentrations of heavy metals detected in this study all exceeded their corresponding background baselines in the soil of Jiangsu Province [34]. The background values were referred from a comprehensive investigation of Chinese soil elements in 1990 by China National Environmental Monitoring Centre. Compared with other freshwater lakes, different metals showed distinguished rankings but the concentrations of heavy metals were generally moderate levels in Lake Yangcheng [4,18,23,25,30]. According to the metal contamination evaluated by PLI and RI, it seemed to be moderately polluted by metals in the entire lake.

According to the spatial distributions of metal concentration, PLI and RI, higher ecological risks were mainly observed in the west part of this lake, where the majority of connected rivers flow into Lake Yangcheng. Therefore, we can speculate that the inflowing rivers carried plenty of metal pollution to the lake and the first step of management strategy should be controlling the external loading. The seasonal variations of heavy metals received less attention in the previous studies due to their non-biodegradability by microbes and higher absorption in the sediments. However, the temporal significance was observed for several kinds of metals in our study, including Cr, Hg, Cd, Zn, and As. The majority of heavy metal concentrations were highest in winter, while the maxima of Hg concentrations were observed in the autumn. Trace metals were also seemed to have more enrichment in winter and summer in Lake Taihu [39], and metal concentrations in November were significantly higher than those in April in Lake Houguan [38], which were similar with the present results.

Furthermore, the average concentrations of heavy metals in the deep sediment were greater than that in the surface sediment. The majority of metal levels increased from the surface to deeper layers of sediment cores and similar results were also observed in other studies [1,40]. Surface sediment are more chemically and biologically active than deep sediment layers and serve as a habitat for benthic organisms. Heavy metals are non-destructible and will often naturally accumulate in soils rather than attenuate [3]. Remarkably, RI value ranged from 366.72 to 371.72 in the deep sediment with an obvious increase. Such results were also recorded in other studies [3,26]. These findings may further support the retention and accumulation of heavy metals in the lake sediment, suggesting more ecological risks posed by metal pollution over time.

### 4.2. Risk Assessment of Heavy Metals in the Sediment

Each heavy metal contamination in the sediment was assessed by CF, I_geo_, and Er, respectively. CF assessment revealed that Hg and Sb posed a considerable risk. According to I_geo_ values, the surface sediment was moderately to heavily polluted by Sb and moderately polluted by Hg, Cd, Ni, and Cu. In terms of Er, Hg showed a considerable contamination; additionally, Cd and Sb exhibited a moderate degree. Overall, among all the selected metals, the most considerable potential ecological risks to aquatic organisms were posed by Hg, Cd, and Sb in the sediment. Additionally, the toxicity of these three metals were very high, with risk coefficients of 40 for Hg, 30 for Cd, and 10 for Sb (similar with As). Generally, Hg, Cd, and Sb belong to non-essential elements, which are more likely to cause harm to the human body than essential elements [41]. People who are exposed to drinking water contaminated by Sb easily obtain damage in the stomach, liver, and kidney [42]. The Sb contamination in Lake Yangcheng received considerable attention in 2014–2015, because Sb concentrations in the water column exceeded the baseline many times, directly threatening the safety of drinking water resources.

The RI value with an average of 285.01 suggested a moderate contamination in the entire lake with most of the contributions posed by Hg, Cd, and Sb. The high contributions of Cd and Hg to RI were similar to many other lakes [25,38,43]. Generally, As, Cd, and Hg are suggested to be the most concerning metals with respect to environmental monitoring and management in Lake Yangcheng due to their high contamination levels and potential eco-risks.

### 4.3. Source Appointment of Heavy Metals in the Sediment

Significant positive correlations between Zn, Cr, Cu, Ni, Cd, and Pb indicated that these elements possibly originated from similar sources [7,44]. The source apportionment indicated that agricultural activities and industrial and municipal sewage were suitable explanations of metal contamination in Lake Yangcheng [20,23]. The lake watershed covers an area of 614.45 km^2^, with the major land uses of residential, manufacture, and cropland. Since the 1990s, the anthropogenic fluxes of heavy metals began to increase, concurrent with the economic growth and development in the western Lake Taihu Basin after the Chinese economic reform [45]. Due to the rapid development of economy in Suzhou city, structural pollution became dominant in the catchment, with chemical, textile, printing and dyeing, machinery manufacturing, and cement plants as major pollution sources. The land use in the catchment changed from farm land to industrial land rapidly. Cd is usually associated with intensive application of fertilizer and pesticide in the farm land around the lake catchment. With the progress of urbanization, the population may have a sudden increase, resulting in more wastewater discharges. The major source of Sb pollution was the sewage wastewater from industries. These discharges were carried into the lake by runoffs, leading to the excessive Sb in the water and lake sediment.

Lake Yangcheng and its connected rivers are the main water sources of adjacent aquaculture areas [41]. More pesticides and fertilizers were used by farmers to increase production. The Chinese mitten crabs (Eriocheir Sinensis) easily accumulate heavy metals in their bodies and female crabs are more likely to accumulate Hg and Cd during growth. Heavy metals can accumulate in the ecosystems and the majority are toxic to aquatic organisms. The occurrence of high concentrations of Hg, Cd, and Sb may pose a potential ecological risk from long-term consumption. Therefore, it is urgent to apply a practical method to remove abundant metals from the sediment, such as sediment dredging and the use of environmentally friendly washing agents [46].

## 5. Conclusions

In summary, this study explored the spatial distribution of nine heavy metals in the surface sediment of Lake Yangcheng and gave a comprehensive evaluation of metal contamination using different pollution indices. The average concentrations of heavy metals in the surface sediment were generally higher than their corresponding background values in this shallow eutrophic lake. Strong fluctuations were observed in heavy metal levels with increasing sediment depth. The vertical distribution of heavy metals further supported historical accumulation and resistant degradation in the deep sediment. In terms of the spatial distribution of heavy metals, similar patterns were observed for the majority of metals with higher values in the east lake, suggesting more metal enrichment and greater ecological risks in this specific lake area. The temporal patterns of metal concentration, PLI, and RI indicated more enrichment in the winter and summer. The calculated CF, I_geo_, and Er of each heavy metal in the sediment showed various degrees of pollution, highlighting that Hg and Sb would pose a considerable potential ecological risk. The sources of the heavy metals were also discussed based on correlation matrix analysis and PCA. Zn, Cr, Cu, Ni, Cd, and Pb were in the same group, indicating a mixed source comprising both lithogenic and human activities. The sources of Hg, As, and Sb were categorized as anthropogenic. This study attempted to provide some support for the control and management of heavy metal pollution in freshwater lakes.

## Figures and Tables

**Table 1:** The averages (unit: mg/kg) and standard deviation (SD) of heavy metal concentrations, PLI, and RI in different seasons. Significant *p* values are in bold.

Metals	Winter	Spring	Summer	Autumn	*p*
Pb	31.76 ± 5.20	28.05 ± 4.29	30.46 ± 3.95	30.23 ± 6.92	0.146
Cr	94.91 ± 23.61	85.38 ± 22.26	84.43 ± 15.45	64.13 ± 20.89	**0.006**
Hg	0.070 ± 0.024	0.081 ± 0.050	0.090 ± 0.030	0.121 ± 0.042	**0.001**
Cd	0.36 ± 0.15	0.34 ± 0.12	0.33 ± 0.11	0.24 ± 0.18	**0.038**
Ni	67.89 ± 19.88	65.46 ± 16.45	60.44 ± 12.08	45.31 ± 17.54	0.593
Cu	60.02 ± 44.42	55.53 ± 41.50	50.75 ± 31.07	52.38 ± 39.65	0.150
Zn	154.01 ± 81.02	141.97 ± 74.10	135.27 ± 58.66	111.67 ± 66.55	**0.014**
As	15.73 ± 1.69	13.04 ± 1.96	12.73 ± 2.07	13.05 ± 5.91	**0.011**
Sb	4.83 ± 1.28	4.72 ± 1.46	4.30 ± 1.05	3.68 ± 1.03	0.077
PLI	2.13 ± 0.55	2.01 ± 0.51	1.97 ± 0.32	1.72 ± 0.64	0.172
RI	276.7 ± 78.95	282.23 ± 102.44	285.96 ± 54.02	285.01 ± 71.18	0.67

**Table 2:** The average concentrations of heavy metals in the surface sediment of Lake Yangcheng and comparisons with other freshwater lakes (unit: mg/kg).

Lake	Pb	Cr	Hg	Cd	Ni	Cu	Zn	As	Sb	Reference
Background value	26.2	77.8	0.03	0.126	26.7	22.3	62.6	10	1.04	[34]
West area	33.70	104.22	0.107	0.407	75.05	94.75	213.50	13.47	3.53	This study
Middle area	30.67	84.01	0.081	0.290	65.16	47.59	123.78	13.21	4.71	This study
East area	27.37	66.34	0.085	0.274	46.00	32.67	91.85	14.03	4.73	This study
Yangcheng	30.12	82.21	0.090	0.316	59.77	54.67	135.73	13.64	4.38	This study
Nansihu	14.43	88.21	-	0.134	43.86	28.06	83.94	-	-	[2]
Poyang	50.4	135.9	-	0.7	-	62.0	132.9	-	-	[18]
Taihu	29.70	68.85	0.145	0.610	36.19	35.53	109.32	16.99	-	[30]
Chaohu	47.1	72.5	0.114	0.44	-	26.0	137.8	10.4	-	[4]
Baiyangdian	44.16	55.81	-	0.73	-	39.20	126.88	14.73	-	[23]
Erhai	47.4	103.8	0.167	1.10	52.2	63.1	109	26.9	-	[25]
Dongping	35.5	89.3	0.055	0.285	-	52.0	100.5	25.3	-	[37]
Houguan	39.3	76.3	0.393	2.68	-	38.6	90.7	30.0	-	[38]

**Table 3:** The Spearman correlation coefficients (r) for the relationships between heavy metals in Lake Yangcheng (*n* = 56). ** and * indicate significant correlation at the 0.01 level (2-tailed) and at the 0.05 level (2-tailed), respectively.

	Pb	Cr	Hg	Cd	Ni	Cu	Zn	As	Sb
Pb	1								
Cr	0.627 **	1							
Hg	0.373 **	0.020	1						
Cd	0.668 **	0.642 **	0.207	1					
Ni	0.573 **	0.946 **	0.036	0.673 **	1				
Cu	0.645 **	0.860 **	0.178	0.624 **	0.894 **	1			
Zn	0.650 **	0.947 **	0.134	0.681 **	0.938 **	0.930 **	1		
As	0.128	0.176	−0.095	0.160	0.119	0.134	0.098	1	
Sb	0.259	0.039	−0.114	0.310 *	0.033	−0.133	−0.062	0.124	1

**Table 4:** Principal component analysis for heavy metals detected in the surface sediments of Lake Yangcheng.

	Initial Eigenvalues			Heavy Metals	Component	
	Total	Variance %	Cumulative %		PC1	PC2
1	4.977	55.298	55.298	Pb	0.751	0.368
2	1.59	17.664	72.961	Cr	0.932	0.09
3	0.971	10.793	83.754	Hg	0.486	−0.509
4	0.813	9.028	92.782	Cd	0.87	0.257
5	0.304	3.374	96.157	Ni	0.89	0.178
6	0.204	2.27	98.427	Cu	0.897	−0.386
7	0.09	0.996	99.423	Zn	0.965	−0.186
8	0.042	0.465	99.888	As	0.161	0.228
9	0.01	0.112	100	Sb	−0.007	0.924
				Eigenvalues	4.977	1.59
				% of Variance	55.298	17.664
				% of Cumulative	55.298	72.961

## Data Availability

All relevant data is within the manuscript.

## Supplementary Materials

The following are available online at https://www.mdpi.com/article/10.3390/toxics10010016/s1, Figure S1: The vertical distributions of RI in the deep sediment according to the values of 14 sampling sites in Lake Yangcheng, Figure S2: The seasonal variations and spatial distributions of PLI in the surface sediment according to the values of 14 sampling sites in Lake Yangcheng. The colored areas indicate index values at the sampling sites. The color spectrum ranges from green (lowest) to red (highest), Figure S3: The seasonal variations and spatial distributions of RI in the surface sediment according to the values of 14 sampling sites in Lake Yangcheng. The colored areas indicate index values at the sampling sites. The color spectrum ranges from green (lowest) to red (highest).
