# Supplementary material for: Spatial–Temporal Variations, Ecological Risk Assessment, and Source Identification of Heavy Metals in the Sediments of a Shallow Eutrophic Lake, China
Source: Toxics. 2022 Jan 4;10(1):16. doi: 10.3390/toxics10010016 (PMC8778156; doi:10.3390/toxics10010016)
Supplement: Supplementary file 1 [file toxics-10-00016-s001.zip › toxics-1501214-supplementary.pdf]

toxics

Supplementary Materials: Spatial-temporal variations, ecological risk assessment, and source identification of heavy metals in the sediments of a shallow eutrophic lake, China

Xiaomei Su, Hong Ling, Dan Wu, Qingju Xue and Liqiang Xie

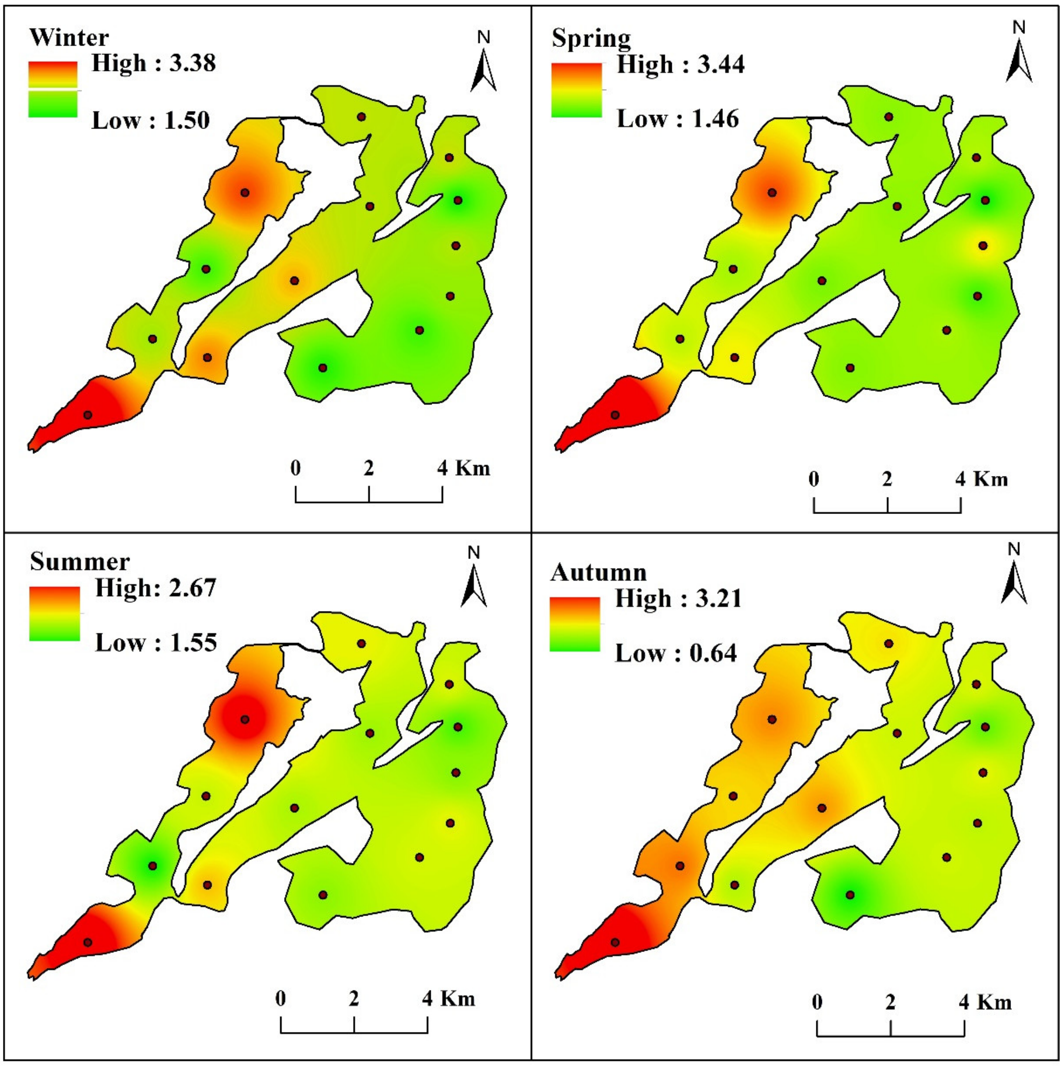

Figure S1. The seasonal variations and spatial distributions of PLI in the surface sediment according to the values of 14 sampling sites in Lake Yangcheng. The colored areas indicate index values at the sampling sites. The color spectrum ranges from green (lowest) to red (highest).

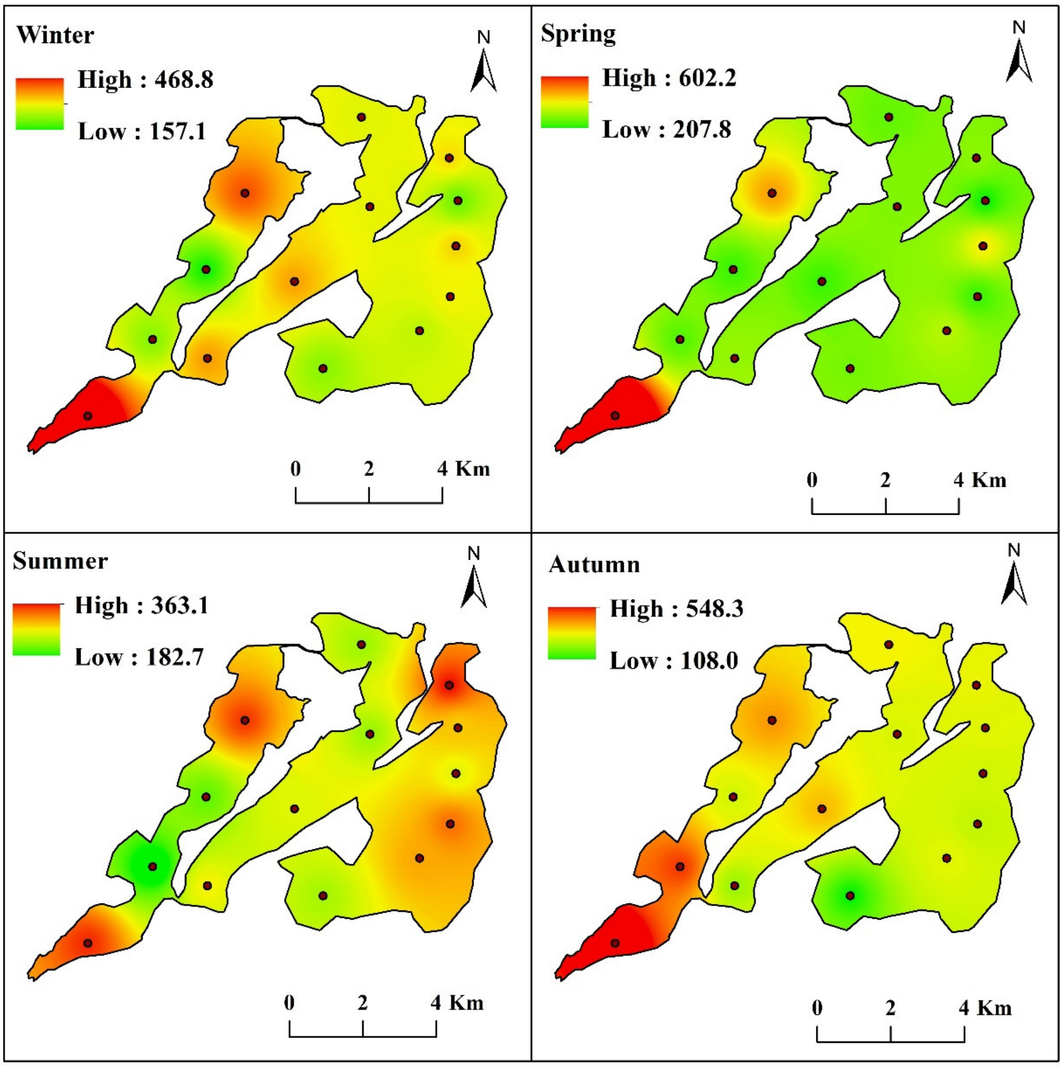

Figure S2. The seasonal variations and spatial distributions of RI in the surface sediment according to the values of 14 sampling sites in Lake Yangcheng. The colored areas indicate index values at the sampling sites. The color spectrum ranges from green (lowest) to red (highest).

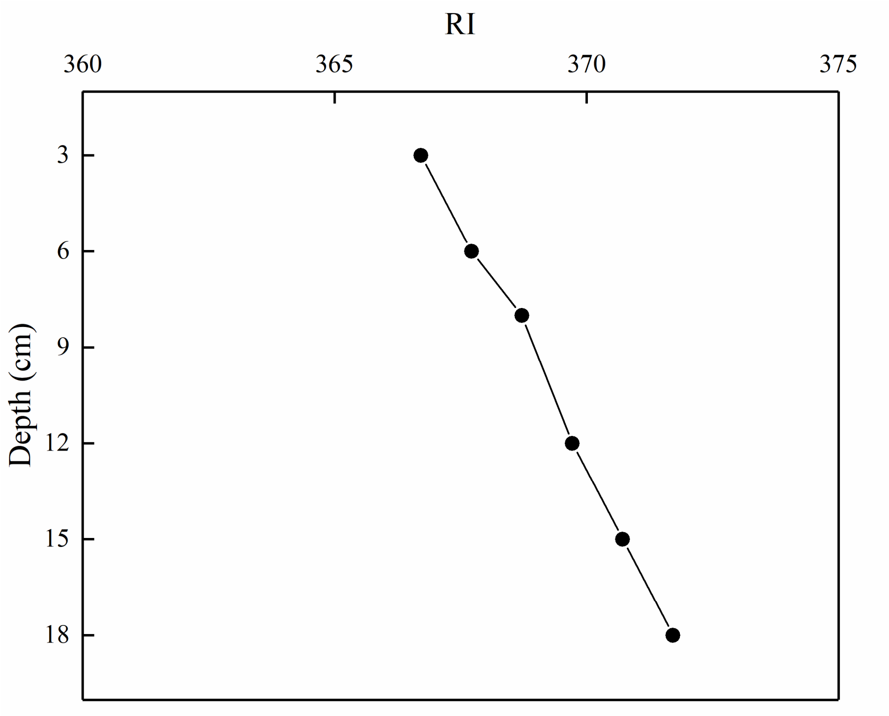

Figure S3. The vertical distributions of RI in the deep sediment according to the values of 14 sampling sites in Lake Yangcheng.
